# Occurrence of *Dirofilaria repens* in wild carnivores in Poland

**DOI:** 10.1007/s00436-023-07823-5

**Published:** 2023-03-20

**Authors:** Mustafa Alsarraf, Dorota Dwużnik-Szarek, Joanna Hildebrand, Ewa Julia Mierzejewska, Agnieszka Kloch, Kamila Kot, Korneliusz Kurek, Sabina Nowak, Robert W. Mysłajek, Izabella Myśliwy, Marcin Popiołek, Anna Rodo, Mohammed Alsarraf, Katarzyna Tołkacz, Mariia Topolnytska, Dagmara Wężyk, Anna Bajer

**Affiliations:** 1 Department of Eco-epidemiology of Parasitic Diseases, Institute of Developmental Biology and Biomedical Sciences, Faculty of Biology, University of Warsaw, Miecznikowa 1, 02-096 Warsaw, Poland; 2 Department of Parasitology, University of Wrocław, Przybyszewskiego 63, Str, 51-148 Wrocław, Poland; 3 Department of Ecology, Institute of Functional Biology and Ecology, Faculty of Biology, University of Warsaw, Biological and Chemical Research Centre, Żwirki i Wigury, 101 Warsaw, Poland; 4 Masurian Centre for Biodiversity Research and Education, Faculty of Biology, University of Warsaw, Urwitałt 1, 11-730 Mikołajki, Poland; 5 Department of Pathology and Veterinary Diagnostics, Faculty of Veterinary Medicine, Warsaw University of Life Sciences-SGGW, 159c Nowoursynowska Street, 02-766 Warsaw, Poland; 6 Institute of Biochemistry and Biophysics, Polish Academy of Sciences, Pawińskiego 5A, 02-106 Warsaw, Poland

**Keywords:** *Dirofilaria repens*, Red fox, Grey wolf, Eurasian badger, Pine marten, Stone marten, Raccoon dog, Raccoon, Molecular detection

## Abstract

*Dirofilaria repens* is an expanding vector-borne zoonotic parasite of canines and other carnivores. Sub-clinically infected dogs constitute the most important reservoir of the parasite and the source of infection for its mosquito vectors. However, occurrence of *D. repens* infection in wild animals may contribute to the transmission of the parasite to humans and may explain the endemicity of filariae in newly invaded regions. The aim of the current study was to determine the occurrence of *D. repens* in 511 blood and spleen samples from seven species of wild carnivores (wolves, red foxes, Eurasian badgers, raccoons, raccoon dogs, stone martens, and pine martens) from different regions of Poland by means of a PCR protocol targeting the 12S rDNA gene. *Dirofilaria repens*–positive hosts were identified in seven of fourteen voivodeships in four of the seven regions of Poland: Masovia, Lesser Poland, Pomerania and Warmia-Masuria. The highest prevalence was found in Masovia region (8%), coinciding with the highest previously recorded prevalence in dogs in Central Poland. The DNA of *Dirofilaria* was detected in 16 samples of three species (total prevalence 3.13%). A low and similar percentage of positive samples (1.9%, 4.2% and 4.8%) was recorded among badgers, red foxes, and wolves, respectively. *Dirofilaria repens*–positive hosts were identified in seven of fourteen voivodships. Based on detection in different voivodeships, *D. repens*–positive animals were recorded in four out of the seven regions of Poland: in Masovia, Lesser Poland, Pomerania, and Warmia-Masuria. The highest prevalence of filariae was found in Masovia region (8%), reflecting the highest previously recorded prevalence in dogs (12–50%) in Central Poland. In summary, we conducted the first comprehensive study on the epidemiology of *D. repens* in seven species of wild hosts in all seven regions of Poland and identified the first case of *D. repens* infection in Eurasian badgers in Poland and the second in Europe.

## Introduction

*Dirofilaria repens* is an important vector-borne parasite of canines and other carnivores that is transmitted by mosquitos of the genera *Culex*, *Aedes*, and *Anopheles* (Capelli et al. 2018; Shaikevich et al. 2019). Furthermore, some invasive mosquito species may also act as competent vectors for *Dirofilaria* spp. as, for example, *Aedes koreicus* which is now considered a new invasive species in Europe (Montarsi et al. 2015). This parasite usually causes asymptomatic subcutaneous infections in canines and is of zoonotic importance because of its ability to infect humans (Petry et al. 2015; Capelli et al. 2018). In humans, the worms can localize in the peri-orbital region or in subcutaneous nodules (Klintebjerg et al. 2015).

In dogs and wild canids, *D. repens* occurs in several forms: as maturing or adult parasites inhabiting subcutaneous and connective tissues and as a first larval stadium, microfilariae (MF), occurring in circulating blood. Dirofilariosis in dogs can cause diverse clinical signs, due to the different locations of adult and larval stages (Deak et al. 2021; Osińska et al. 2014; Tarello et al. 2011). For example, nematodes migrating under the skin can sporadically cause erythema, papules, alopecia, and pruritus (Tarello et al. 2011). Fatal infection with *D. repens* is rare but may occur when a massive microfilarial burden is present in the host circulation and internal organs, resulting in multi-organ failure (Mircean et al. 2017; Osińska et al. 2014).

In Europe, the disease is spreading from the historically endemic Mediterranean countries towards previously *Dirofilaria*-free central and northern countries including Austria, Germany, Netherlands, Poland, Latvia, Lithuania, Estonia, and Finland (Sałamatin et al. 2013; Bajer et al. 2016; Fuehrer et al. 2016; Capelli et al. 2018; Alsarraf et al. 2021; Fuehrer et al. 2021).

Although subcutaneous dirofilariosis is widely studied in pets, it is poorly investigated in wild carnivores such as red foxes (*Vulpes vulpes*), grey wolves (*Canis lupus*), Eurasian badgers (*Meles meles*), raccoon dogs (*Nyctereutes procyonoides*), stone martens (*Martes foina*), pine martens (*Martes martes*), and raccoons (*Procyon lotor*) (Magi et al. 2008; Otranto et al. 2015).

Studies from Uzbekistan, Serbia, Germany, Macedonia, Russia (Krasnodar Krai), Romania, and Iraq have shown that wild carnivores such as red foxes, golden jackals (*Canis aureus*) ,and grey wolves can be infected and, hence, can contribute to the endemic occurrence of the parasite (Cirovic et al. 2014; Kravchenko et al. 2016; Härtwig et al. 2016; Ionică et al. 2017; Otranto et al. 2019; Potkonjak et al. 2020; Safarov et al. 2021).

In comparison to the relatively few studies on reservoir hosts of *D. repens*, the occurrence of *Dirofilaria immitis* has been widely studied in wildlife and reported frequently in a wide range of carnivorous species including grey wolves, red foxes, raccoon dogs, golden jackals, wild cats, and domestic ferrets (Gomes-de-Sá et al. 2022; Hiedari et al. 2015; Penezić et al. 2014; Kido et al. 2011; Villanueva-Saz et al. 2022; Moroni et al. 2020).

The existence of wildlife infected with *D. repens* constitutes a risk factor for both pet and human populations since the loss of their habitats has forced wild animals to invade urban areas and poses a risk for transmission to both humans and their pets via the vector hosts (Simón et al. 2012).

Poland is considered to be a newly endemic region for *D. repens* due to the several autochthonous dirofilariasis cases that were reported in humans between 2009 and 2018 (Cielecka et al. 2012; Borkowski et al. 2015; Kłudkowska et al. 2018; Kołodziej et al. 2019), especially in Central Poland, where prevalence of *D. repens* in dogs has been reported to be 12% (Alsarraf et al. 2021).

To date, there have been no comprehensive epidemiological studies on the prevalence of *D. repens* in wild carnivores in Poland. This lack of studies is partly due to the difficulty of collecting fresh blood samples from free-living animals or their corpses but especially because some of these animals are protected species (e.g., grey wolves). However, raccoons and raccoon dogs are known to be invasive species in Europe including Western Poland and are competent hosts for a range of blood and intestinal parasites (Myśliwy et al. 2022; Hildebrand et al. 2022, 2022). Hence, their spread to new regions in Europe may facilitate the spread of parasites for which they constitute competent hosts.

We hypothesized that *D. repens* should be present in free-living carnivores in Poland because this parasite has existed in the last decade and is now well established in dog populations in the region.

The aim of this study is to estimate the prevalence of *D. repens* in seven different species of wild carnivores (grey wolves, red foxes, Eurasian badgers, raccoons, raccoon dogs, stone martens, and pine martens) collected from different regions in Poland.

## Materials and methods

### Sample collection

A total of 511 blood and spleen samples were collected between 2016 and 2022 from seven animal species (287 red foxes, 62 grey wolves, 54 Eurasian badgers, 58 raccoons, 35 raccoon dogs, 8 stone martens, and 7 pine martens). Samples were collected from carcasses obtained by the authors from hunters during hunting seasons (Dwużnik et al. 2020; Hildebrand et al. 2022, 2022), from animals killed or injured in car accidents, and individuals captured for telemetry studies or found dead from unknown reasons in the forests, roads, and fields (Szewczyk et al. 2019).

Blood from the heart was collected from most animals (*N* = 369) including grey wolves, red foxes, raccoon dogs, pine martens, Eurasian badgers, and raccoons and was stored in 0.001M EDTA at a temperature of − 20 °C. The spleen was dissected from 155 animals including red foxes, stone martens, Eurasian badgers, pine martens, raccoons, and raccoon dogs and stored at − 20 °C. Spleen samples were used in our study, because they had been used previously for the monitoring of blood parasites in wildlife (Mierzejewska et al. 2021), including studies on *Dirofilaria* spp. (Härtwig et al. 2016). In a histopathological study in dogs, numerous MF were found in microvessels and tissues of internal organs (Osińska et al. 2014), supporting the use of tissues from solid organs as material suitable for epidemiological research.

**Fig. 1:**
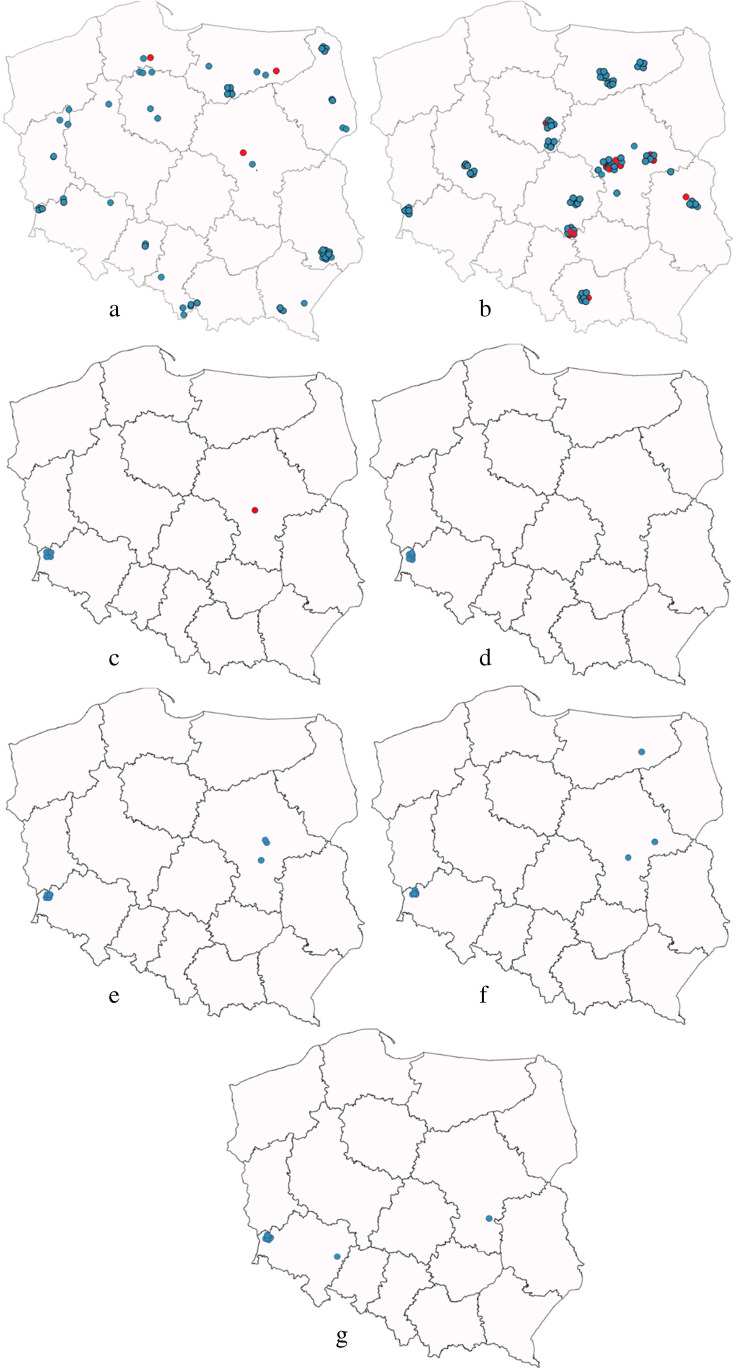
Collection sites for *Canis lupus* (**a**), *Vulpes vulpes* (**b**), *Meles meles* (**c**), *Procyon lotor* (**d**), *Martes martes* (**e**), *Nyctereutes procyonoides* (**f**), and *Martes foina* (**g**). Blue dots represent the collection site of the non-infected animals while the red dots represent *D. repens*–positive samples

Samples originating from the area of 14 voivodeships (major administrative units of Poland marked on Fig. 1) were assigned to seven geographical regions of Poland. Most samples originated from Lower Silesia and Masovia voivodships (Table 1).

**Table 1:** Prevalence of *D. repens* by region, voivodeship, and host species

Prevalence of *D. repens*										
Region	Voivodeship	Red foxes	Wolves	European badgers	Beech martens	Pine martens	Raccoons	Raccoon dogs	Total prevalence (region)	Total prevalence (voivodeship)
Greater Poland	Greater Poland	0% (0/9)	0% (0/1)	-	-	-	-	-	0% (0/15)	0% (0/10)
	Lubusz	-	0% (0/5)	-	-	-	-	-		0% (0/5)
Lesser Poland	Lesser Poland	7.7% (1/13)	0% (0/1)	-	-	-	-	-	5% (2/40) 95% CI: 0.6–17%	7.1% (1/14) 95% CI: 0.2–30%
	Subcarpathian	-	0% (0/4)	-	-	-	-	-		0% (0/4)
	Lublin	16.7% (1/6)	0% (0/16)	-	-	-	-	-		4.5% (1/22) 95% CI: 0.1–23%
Masovia	Masovian	7.4% (6/81)	50% (1/2)	100% (1/1)	0% (0/1)	0% (0/3)	-	0% (0/2)	8.4% (11/131) 95% CI: 4–15%	8.9% (8/90) 95% CI: 4–17%
	Łódź	7.3% (3/41)	-	-	-	-	-	-		7.3% (3/41) 95% CI: 2–20%
Podlachia	Podlaskie	-	0% (0/8)	-	-	-	-	-	0% (0/8)	0% (0/8)
Pomerania	Pomeranian	-	50% (1/2)	-	-	-	-	-	2.9% (2/70) 95% CI: 0.4–10%	50% (1/2) 95% CI: 0.01–99%
	Kuyavian-Pomeranian	1.6% (1/63)	0% (0/5)	-	-	-	-	-		1.5% (1/8) 95% CI: 0.3–52%
Silesia	Lower Silesia	0% (0/41)	0% (0/6)	0% (0/53)	0% (0/7)	0% (0/4)	0% (0/58)	0% (0/31)	0% (0/208)	0% (0/200)
	Silesian	-	0% (0/6)	-	-	-	-	-		0% (0/6)
	Opole	-	0% (0/2)	-	-	-	-	-		0% (0/2)
Warmia-Masuria	Warmian-Masurian	0% (0/33)	10% (1/10)	-	-	-	-	0% (0/2)	2.2% (1/45) 95% CI: 0.06–12%	2.2% (1/45) 95% CI: 0.06–12%
Total prevalence (species)		4.2% (12/287) 95% CI: 2–7%	4.8% (3/62) 95% CI: 1–13%	1.9% (1/54) 95% CI: 0.05–9.8%	0% (0/8)	0% (0/7)	0% (0/58)	0% (0/33)	Total prevalence in the study 3.1% (16/511) 95% CI: 1.9–5%	

A significant number of samples originated from the Bory Dolnośląskie Forest in Lower Silesia voivodeship in the Silesian region. From this region, carcasses were provided by licensed hunters during the autumn/winter hunting seasons or in the course of the Life Project LIFE11 NAT/PL/428 “Active protection of lowland populations of capercaillie (*Tetrao urogallus*) in the Bory Dolnośląskie Forest and Augustowska Primeval Forest.” Raccoons were shot in Western Poland (Lower Silesia) as the part of a control program of invasive species Hildebrand et al. 2022, 2022).

### DNA extraction and molecular detection

Genomic DNA was extracted from blood and spleen samples using Genomic Mini AX Tissue Spin (A&A Biotechnology, Gdańsk, Poland) following the manufacturer’s protocol. A 327 bp fragment of 12S rDNA was amplified for the detection of *D. repens* as described by Gioia et al. (2010).

Amplicons were visualized with Midori Green stain (Nippon Genetics Europe GmbH, Düren, Germany) following electrophoresis in 1.5% agarose gels. Selected amplicons from foxes and all PCR products from other host species were sequenced by a private company (Genomed S.A., Warsaw, Poland).

Sequences were edited using CodonCode Aligner 10.0.2 (CodonCode Corporation, Centerville, MA, USA). The resulting sequences were compared with sequences deposited in GenBank NCBI.

### Statistical analysis

Statistical analysis of prevalence was conducted using the maximum likelihood method based on the log-linear analysis of contingency tables in the IBM SPSS v. 21 software (IBM Corporation).

The factors for analysis included region of animal origin (1–7) or voivodships of animal origin (1–14), host species (1–7), and infection status for *D. repens* (0, not infected; 1, infected). For each level of analysis in turn, beginning with the most complex model, involving all possible main effects and interactions, those combinations that did not contribute significantly to explaining variation in the data were eliminated in a stepwise fashion beginning with the highest-level interaction (backward selection procedure). Prevalence of *D. repens* by voivodeship, region, and host species is provided in Table 1.

## Results

The DNA of *Dirofilaria* was detected in 16 blood samples of three species (total prevalence 3.13%, Table 1). Prevalence ranged between 1.2 and 4.8% (not significant (NS)) among red foxes, wolves, and badgers (Table 1).

Ten out of 16 PCR products encompassing all positive samples from wolves; one positive sample from the badger and six PCR products of sufficient quality from foxes were successfully sequenced. All the obtained 12S rDNA sequences (six from foxes, three from wolves, and one from the badger) were identified as *D. repens*, displaying the highest identity (99–100%) to *D. repens* sequences from dogs and wolves deposited in the GenBank database (KX265088, KY828984).

There were significant differences in the prevalence of *D. repens* between voivodeships and consequently between geographical regions of Poland (Table 1, Fig. 1).

*Dirofilaria repens*–positive animals were identified in seven of fourteen voivodships (Table 1, Fig. 1). The highest prevalence of *D. repens* was observed in Masovian and Łódź voivodeships (Table 1) and in Pomeranian voivodeship because one out of the two samples tested positive there (voivodeship × *D. repens* infection: *χ*^2^ = 29.4, *df* = 13, *P* = 0.006).

Following the differences in occurrence by voivodeships, *D. repens*–positive animals were recorded in four out of the seven regions in Poland: in Masovia, Lesser Poland, Pomerania, and Warmia-Masuria (Table 1, Fig. 1). The highest prevalence of *D. repens* was observed in the Masovia region (Central Poland) while no positive samples were found in Greater Poland, Podlachia, and Silesia (region × *D. repens* infection: *χ*^2^ = 23.8, *df* = 6, *P* < 0.001).

## Discussion

The present study is the first to identify *D. repens* in wild, naturally living hosts in Poland and provides evidence for the occurrence of this parasite in four regions of the country. Interestingly, we detected *D. repens* infection in two species of canids (red fox, grey wolf) and in a Eurasian badger, and the overall prevalence of *D. repens* in free living carnivores was the highest in Central Poland (7–9%) and associated with high prevalence in dogs in this region: 12–50% in different studies. Therefore, the zoonotic risk of *D. repens* in Poland might be better represented by the parasite prevalence in dogs (Demiaszkiewicz et al. 2014; Bajer et al. 2014; Bajer et al. 2016; Alsarraf et al. 2021). The overall prevalence of *D. repens* in wild carnivores was 3.1% in our study which corresponds well with reported prevalences ranging between 1.4 and 10.3% in free-living carnivores from Romania, Serbia, Macedonia, Uzbekistan, and Russia (Krasnodar Krai) (Ionică et al. 2017; Ćirović et al. 2014; Safarov et al. 2021; Kravchenko et al. 2016). In our study, canids, i.e., red foxes and grey wolves, presented with the highest prevalence (4–5%), falling within the range reported previously for these two species, 2.8–9.2% (Ionică et al. 2017; Ćirović et al. 2014; Safarov et al. 2021). Interestingly, the prevalence of *D. repens* in red foxes in the current study was much higher than the prevalence of *D. immitis* in red foxes from Romania (4.2% vs. 0.2%) (Ionică et al. 2017) and similarly, the prevalence of *D. repens* in wolves in our study was higher than the prevalence of *D. immitis* in wolves from Serbia (4.8% vs 1.43%) (Penezić et al. 2014). This suggests that in free-living canids, *D. repens* can be less pathogenic than *D. immitis* and able to cause long-lasting infections. Moreover, despite the fact that our study is dependent on the detection of MF DNA and does not detect prepatent or amicrofilariaemic infection (underestimated total prevalence), it has revealed the relatively high prevalence of *D. repens* in the region, in comparison to similar studies in wolves and foxes elsewhere.

Relatively high prevalence in foxes (above 7%) was noted in Masovia, in Central Poland, in a region where the latest detected prevalence in dogs was 12–13% (Alsarraf et al. 2021. Wężyk et al. 2023). The age effect could have contributed to the discrepancy in prevalence between dogs and foxes. In our recent study in this region, prevalence was lowest in dogs up to 2 years old (9.2%) and higher and similar (15–15.5%) in dogs 2–8 years old and > 8 years old (Alsarraf et al. 2021). No data on age structure of the hunted foxes was available in our study; however, in a previous study (Goszczyński et al. 1989) in Central Poland, about 70% of foxes killed in hunting season (September-March) were below 2 years old, and only 30% were older. In Central Poland, fox cubs are born in early spring (March–April) and fox hunting activities are organized in autumn and winter; therefore, most shot animals belong to age groups that are 0.5–1 and 1.5–2 years old (Goszczyński 1989), which corresponds to only 1–2 seasons of exposure to vectors and explain the generally low prevalence in this game species. No *D. immitis*–positive samples were identified in the present study which reflects well the non-endemic status (yet) of heartworm in Poland (reviewed in Fuehrer et al. 2021). On the other hand, the occurrence of *D. repens* in wild carnivores in Poland provides strong support for the recent recognition of the endemicity of subcutaneous dirofilariosis in the country, following the first detection in dogs and humans between 2009 and 2011 (Fuehrer et al. 2021; Demiaszkiewicz et al. 2009).

Wild carnivores can act as reservoirs of infection for humans and pet dogs despite the relatively low prevalence (3.1%) because of the lack of chemotherapeutic treatment; they provide a constant source of *D. repens* for the mosquito vectors which can then transmit the parasite to humans and dogs. Especially, red foxes which often display synanthropic behavior may have closer contact with domestic animals and mosquito vectors in urban and rural environments. Wildlife reservoirs of zoonotic parasites are hard to control; thus, identification of *D. repens* in three species of free-living carnivores is of public health significance.

Raccoons and raccoon dogs have shown low prevalence for helminths and blood parasites in Poland (Popiołek et al. 2011; Hildebrand et al. 2022, 2022). To the best of our knowledge, *D. repens* has not yet been recorded in these two species. In the recent study of helminths of raccoons in Spain, no subcutaneous nematodes (*Dracunculus* and *Dirofilaria* spp.) were found (Sanjuán et al. 2022). However, North American raccoons are known to be the principal hosts of *Dirofilaria tenuis* that causes heartworm infection in this species and may infect also humans (Parks et al. 2011). Raccoon dogs originated from Asia and have been found to be infected with *D. immitis* in a recent study in Japan with a prevalence of 7.4% (Kido et al. 2011).

In our study, we recorded the first case of *D. repens* infection in a Eurasian badger from Poland (Masovia region) and Central Europe, although *D. repens* was detected for first time in Eurasian badgers in the Krasnodar Krai region in Russia with a prevalence of 10.6%, which suggests that badgers can be reservoir hosts for *D. repens* (Kravchenko et al. 2016).

Although *D. repens* was previously found in stone martens (*M. foina*) in Czechia (Miterpáková et al. 2013), all samples from this study tested negative which could be due to the small sample size.

All samples collected and screened from seven host species in Silesia region in Western Poland (*N* = 208) tested negative for the presence of *D. repens*. The majority of those samples (*N* = 200) were collected from the Bory Dolnośląskie forest near the Polish-German border, and our results correspond well with a similar study from the Brandenburg region in Eastern Germany, in which no *D. repens* was detected among 135 samples from red foxes and raccoon dogs (Härtwig et al. 2016). Thus, the occurrence of *D. repens* may be still questionable in this region of Western Poland.

The present study faced a limitation with regard to the detection of the presence of MF by molecular techniques. Previous studies have shown that the sensitivity of detection by this method is approximately a minimum 4 MF/ml, and this limitation prevents the detection of MF in the prepatent period of infection or, in amicrofilaraemic individuals, is likely to result in some underestimation of the actual prevalence of infection (Gioia et al. 2010).

## Conclusion

Here, we conducted the first comprehensive study on the epidemiology of *D. repens* in seven species of wild hosts in all seven regions of Poland. Furthermore, we recorded the first case of *D. repens* in a Eurasian badger (*M. meles*) in Poland and the second in Europe, after the case in Krasnodar Krai (Russia). Further epidemiological studies should be conducted in Europe on the presence of *D. repens* in free-living hosts to better understand their role as reservoirs of this zoonotic parasite.

## Data Availability

All data is involved in the current paper.
